# Efficacy of Virtual Reality and Exergaming in Improving Balance in Patients With Multiple Sclerosis: A Systematic Review and Meta-Analysis

**DOI:** 10.3389/fneur.2021.773459

**Published:** 2021-12-10

**Authors:** Dario Calafiore, Marco Invernizzi, Antonio Ammendolia, Nicola Marotta, Francesco Fortunato, Teresa Paolucci, Francesco Ferraro, Claudio Curci, Agnieszka Cwirlej-Sozanska, Alessandro de Sire

**Affiliations:** ^1^Physical Medicine and Rehabilitation Unit, Department of Neurosciences, ASST Carlo Poma, Mantova, Italy; ^2^Physical and Rehabilitative Medicine, Department of Health Sciences, University of Eastern Piedmont, Novara, Italy; ^3^Translational Medicine, Dipartimento Attività Integrate Ricerca e Innovazione (DAIRI), Azienda Ospedaliera SS. Antonio e Biagio e Cesare Arrigo, Alessandria, Italy; ^4^Department of Medical and Surgical Sciences, University of Catanzaro “Magna Graecia”, Catanzaro, Italy; ^5^Institute of Neurology, Department of Medical and Surgical Sciences, University of Catanzaro “Magna Graecia”, Catanzaro, Italy; ^6^Department of Medical, Oral and Biotechnological Sciences, G. D'Annunzio University of Chieti-Pescara, Chieti, Italy; ^7^Institute of Health Sciences, College of Medical Sciences of the University of Rzeszow, University of Rzeszow, Rzeszow, Poland

**Keywords:** virtual reality, exergames, multiple sclerosis, balance, rehabilitation, meta-analysis

## Abstract

Multiple sclerosis (MS) is one of the most common causes of neurological progressive disease and can lead to loss of mobility, walk impairment, and balance disturbance. Among several rehabilitative approaches proposed, exergaming and virtual reality (VR) have been studied in the recent years. Active video game therapy could reduce the boredom of the rehabilitation process, increasing patient motivation, providing direct feedback, and enabling dual-task training. Aim of this systematic review was to assess the efficacy of exergaming and VR for balance recovery in patients with MS. PubMed, Scopus, and Web of Science were systematically searched from the inception until May 14, 2021 to identify randomized controlled trials (RCTs) presenting: patients with MS as participants, exergaming and VR as intervention, conventional rehabilitation as comparator, and balance assessment [Berg Balance Scale (BBS)] as outcome measure. We also performed a meta-analysis of the mean difference in the BBS via the random-effects method. Out of 93 records, this systematic review included and analyzed 7 RCTs, involving a total of 209 patients affected by MS, of which 97 patients performed exergaming or VR and 112 patients underwent conventional rehabilitation. The meta-analysis reported a significant overall ES of 4.25 (*p* < 0.0001), showing in the subgroup analysis a non-significant ES of 1.85 (*p* = 0.39) for the VR and a significant ES of 4.49 (*p* < 0.0001) for the exergames in terms of the BBS improvement. Taken together, these findings suggested that balance rehabilitation using exergames appears to be more effective than conventional rehabilitation in patients affected by MS.

## Introduction

Multiple sclerosis (MS) is one of the most common causes of progressive neurological disability among young adults (1). Upper limb impairments, muscle weakness, spasticity, reduced functional performance, and fatigue are common clinical manifestations in patients with MS (2–6). A crucial impairment that might be often showed by patients with MS is balance disturbance, which could result in a higher risk of falling and reduced independence in the activities of daily living (ADLs) (7–10). To overcome these highly disabling issues, different rehabilitative approaches have been proposed so far in the literature (11, 12). In addition to conventional physiotherapy and rehabilitation interventions, technological devices are a promising therapeutic intervention in the complex framework of MS treatment. In this scenario, virtual reality (VR) approaches are suggested as potentially useful tools in several rehabilitative pathological conditions (13). Indeed, VR might enhance the interaction with surrounding artificial environment created to appear similar to the original one, allowing a multisensorial feedback training that might further increase the rehabilitation efficacy. Indeed, human balance control is the results of multiple sensory system inputs, integrated into a complex mechanism of constant reweight and adjustments, as visual, vestibular, and proprioception signals are continuously converted to corrective motor actions (14). Hence, a multisensorial augmented reality might be a particularly effective rehabilitation approach in MS balance impairments.

Moreover, it is provided though a display that can be also head-mounted, with complementary motion tracking devices, sound effects, and eventually end-effectors such as joysticks or sensors able to capture even muscle and brain signals (15). VR has been integrated in several neurological diseases rehabilitative protocols, including patients affected by MS, with promising results (13, 16–18). As a complementary tool of VR in rehabilitation programs (19), patients could also perform exergames, defined as the activity of playing video games that involve physical exertion (20). In the recent years, exergaming has been widely used in several rehabilitative programs and clinical study (21–23). Active video game therapy could reduce the boredom of the rehabilitation process, increasing patient motivation, providing direct feedback, and enabling dual-task training. In this study, commercially available exergames (e.g., Nintendo Wii and Microsoft Kinect) have successfully transformed living rooms into playful training environments for about 10 years (24). Clinical and home trials have been conducted to investigate the effectiveness of Nintendo Wii Fit in patients with MS, focusing on balance and gait improvement, but the results are controversial (25). In the recent years, researchers started to evaluate exergames as a rehabilitation tool for patients with MS (26). Exergaming has proved to be an acceptable, feasible, safe, fun, stimulating, and self-motivating tool (27). However, there is limited evidence of its efficacy among neurological pathologies, in particular, in patients with Parkinson's disease, stroke, and hereditary sensory motor neuropathy (28–30). To the best of our knowledge, few randomized controlled trials (RCTs) investigated the efficacy of exergaming in MS. Therefore, in systematic review and meta-analysis, we sought to evaluate the efficacy of exergames and VR compared with conventional rehabilitation treatment in terms of balance improvement in patients affected by MS.

## Methods

### Search Strategy

PubMed, Scopus, and Web of Science databases were systematically searched for English language articles published from the inception until May 14, 2021, according to each specific thesaurus, following the strategy depicted by Supplementary Table 1. This systematic review with meta-analysis was conducted according to the guidance of the Preferred Reporting Items for Systematic Reviews and Meta-Analyses (PRISMA) guidelines (31) and the Cochrane Handbook for Systematic Reviews of Interventions (32). Systematic review protocol has been registered on the International Prospective Register of Systematic Reviews (PROSPERO) (number: CRD42021257449).

### Selection Criteria

After removing duplicates, two reviewers independently screened all the articles for eligibility. In case of disagreement, a consensus was reached with the opinion of a third reviewer. RCTs were considered eligible, if responding to the questions defined by the following the participants, intervention, comparator and outcome (PICO) model:

P) Participants: patients with MS.
I) Intervention: Exergames and/or VR.
C) Comparator: Conventional rehabilitation.
O) Outcome measure: Balance assessed by the Berg Balance Scale (BBS).

We included only RCTs with two groups (study group and control group) providing data at the end of the intervention (after 1 week later as maximum). We excluded: (1) studies including patients with MS aged <18 years; (2) studies including patients with MS with the Expanded Disability Status Scale (EDSS) score > 6; (3) cross-over study design; (4) studies written in a language different from English; (5) full-text unavailability (i.e., posters and conference abstracts); and (6) studies involving animals.

### Data Extraction

Two reviewers independently extracted main data from the included RCTs through a customized data extraction model on a Microsoft Excel sheet. In case of disagreement, a consensus was obtained asking the opinion of another reviewer.

We extracted the following data: (1) First author; (2) Publication year; (3) Nationality; (4) Age of study participants; (5) Type of exergames and/or VR as intervention; (6) Type of control (placebo or sham treatment); (7) Population and number of patients included in the RCTs; (8) the BBS values as outcome measure; and (9) Main findings.

### Data Synthesis

The RCTs were synthesized describing extracted data and the study quality was independently assessed by two authors according to the PEDro scale (33). In case of disagreement, a third reviewer was asked to obtain a consensus. RCTs included were classified, according to the PEDro scale (33), as excellent (9–10 points), good (6–8 points), fair (4–5 points), or poor (<4 points) quality studies. Additionally, two authors assessed the risk-of-bias using the revised Cochrane risk-of-bias 2 (RoB 2) tool for randomized trials (34) and discussed any disagreements until consensus was reached with a third reviewer.

### Statistical Analysis

The statistical analysis was performed on Stata version 15.0 (Stata, College Station, Texas, USA) and RevMan version 5.3. The heterogeneity among comparisons was estimated by the chi-squared and *I*^2^ statistic tests. An *I*^2^ > 50% determined significant heterogeneity across the articles. Effect size (ES) measure and a random-effects model were obtained to determine the pooled estimates with 95% CIs. Missing means and SDs were estimated from medians, ranges, and interquartile ranges (IQRs) using the method introduced by Hozo et al. (35). Then, we carried out a sensitivity analysis on the stability of the combined results. Lastly, we also performed a subgroup analysis on the intervention type to investigate the source of heterogeneity. The potential publication bias was assessed using a contour-enhanced funnel plot of effect size against its SE.

## Results

### Study Characteristics

**Figure 1:**
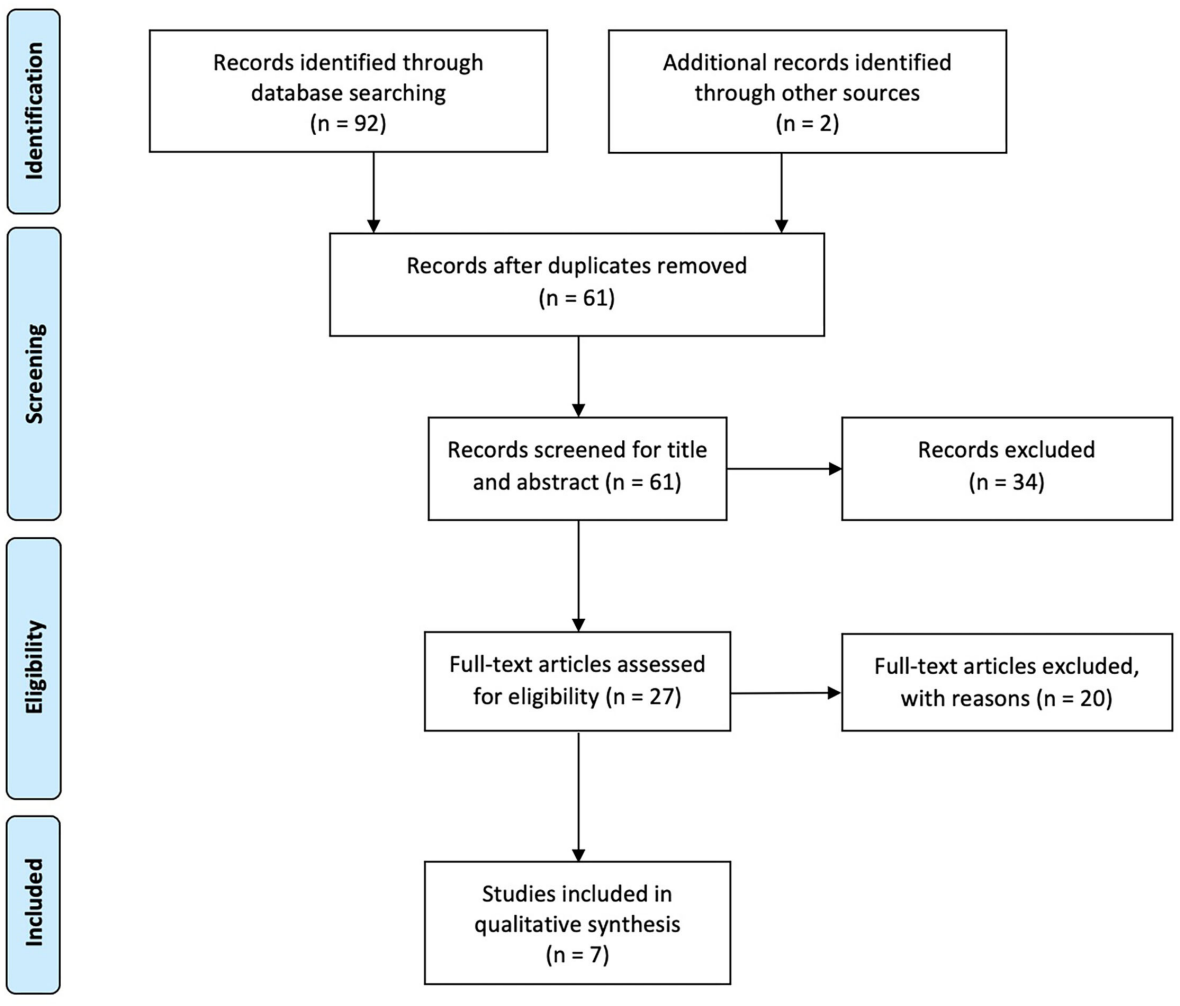
The Preferred Reporting Items for Systematic Reviews and Meta-Analyses (PRISMA) flow diagram.

At the end of the search, 93 studies were identified, 61 of which were considered suitable for title and abstract screening, after the removal of duplicates. Out of these, 34 studies were excluded after the title and abstract screening, according to the PICO model. Thus, the selected articles were assessed for eligibility and 20 of them were excluded according to the following reasons: not intervention of interest (*n* = 3), not comparison of interest (*n* = 6), and not outcome of interest (*n* = 11) (see Table 1 for further details). Therefore, 7 RCTs (26, 36–41) were included in this systematic review, as shown by the PRISMA flowchart in Figure 1. The main characteristics of these studies are given in detail in Table 2. The included studies (26, 36–41) have been published in the last 7 years (from 2003 to 2020). Five (36, 37, 39–41) (71.4%) studies were conducted in Europe [one (36) study from Italy, two (37, 41) study from Spain, one (39) study from Hungary, one (40) study from Israel] and two (26, 38) (28.6%) studies were conducted in Eastern Mediterranean [one (26) study from Jordan, one (38) study from Iran]. A total of 209 subjects were analyzed and 97 subjects performed VR or exergaming as balance training, whereas 112 subjects were included in the control group (undergoing conventional balance training). Study cohorts of the RCTs included ranged from 11 (41) to 47 (37) patients, with a mean age ranging from 34.9 ± 8.9 (26) to 48.3 ± 10.8 years (41). Concerning the follow-up evaluations, only one RCT (38) performed a follow-up at 12 weeks from baseline. Five RCTs (26, 36–39) investigated the effectiveness of exergaming and two RCTs (40, 41) investigated the effectiveness of VR.

**Table 1:** Reasons for article exclusion by the present systematic review.

Articles excluded after title and abstract screening phase (***n*** = 34)*	
Not population of interest	0 (0.0)
Not intervention of interest	0 (0.0)
Not comparison of interest	2 (5.8)
Not outcome of interest	1 (2.9)
Study design different from RCTs	30 (88.2)
Language different than English	1 (2.9)
**Articles excluded after full-text screening phase (***n*** = 20)**	
Not population of interest	0 (0.0)
Not intervention of interest	3 (15.0)
Not comparison of interest	6 (30.0)
Not outcome of interest	11 (55.0)
Full-text unavailability	0 (0.0)
Language different than English	0 (0.0)
Simultaneous publication in two scientific Journals	0 (0.0)

*The exclusion of the articles followed the PICO model defined in the Methods Section. Data are expressed as counts (percentages)*.

* *Papers were excluded also for more than one reason during the title and abstract screening phase*.

**Table 2:** Main characteristics of the randomized controlled trials included in the present systematic review.

**Article**	**Nationality**	**Study group**	**Control group**	**EDSS**	**Intervention**	**Comparison**	**Outcome measure and time-point assessments**	**Main findings**
Brichetto et al. (36) Mult Scler 2013	Italy	*n =* 18; 8 M/10 F Age: 40.7 ± 11.5 years	*n =* 18; 6 M/12 F Age: 43.2 ± 10.6 years	≤ 6	Wii^®^ balance board, 12 sessions, 3 times/week	Conventional balance training, 12 sessions, 60 min, 3 time/week	BBS at baseline and at the end of treatment	Significant differences in BBS between groups in favor of experimental group (*p* < 0.05)
Gutierrez et al. (37) NeuroRehabilitation 2013	Spain	*n =* 24; 11 M/13 F Age: 39.7 ± 8.1 years	*n =* 23; 9 M/14 F Age: 42.8 ± 7.4 years	Ranging from 3 to 5	Xbox360^®^ console with Microsoft^®^ Kinect, 40 sessions, 4 time/week	Conventional balance training, 2 times/week, 40 min	BBS at baseline and at the end of treatment	Significant differences in BBS between groups in favor of experimental group (*p* < 0.001)
Khalil et al. (26) NeuroRehabilitation 2018	Jordan	*n =* 16; 4 M/12 F Age: 39.9 ± 12.8 years	*n =* 16; 6 M/10 F Age: 34.9 ± 8.9 years	Ranging from 3 to 6.5	Wii ^®^ balance board, 12 sessions + 6 session at home, 3 times/week	Conventional home balance training, 18 sessions, 3 times/week	BBS at baseline and at the end of treatment	Significant differences in BBS between groups in favor of experimental group (*p* < 0.012)
Mohlemi F et al. (38) Arch Phys Med Rehabil 2020	Iran	*n =* 19; 7 M/12 F Age: 36.8 ± 8.4 years	*n =* 20; 8 M/12 F Age: 41.6 ± 8.4years	<6	Xbox360^®^ with Microsoft's Kinect + conventional balance training, 18 sessions, 3 time/week	Conventional balance training, 18 sessions, 3 time/week	BBS at baseline, at the end of treatment and after 3 months	No significant differences in BBS between groups were found
Tollar et al. (39) Med Sci Sport Exerc. 2020	Hungary	*n =* 14; 7 M/7 F Age: 48.2 ± 5.9 years	*n =* 14; 7 M/7 F Age: 46.9 ± 6.4 years	Ranging from 4 to 6	Xbox360 ^®^ with Microsoft's Kinect, 25 sessions, 5 times/week	Conventional balance training, 25 sessions, 5 time/week	BBS at baseline and at the end of treatment	No significant differences in BBS between groups were found
Karlon et al. (40) J Neuroeng Rehabil 2016	Israel	*n =* 15; 5 M/10 F Age: 47.3 ± 9.6 years	*n =* 15; 6 M/9 F Age: 43.9 ± 10.6 years	Ranging from 3 to 6	Immersive VR, CAREN system, 12 sessions, 30 minutes, 2 times/week	Conventional balance training, 12 sessions, 30 minutes, 2 times/week	BBS at baseline and at the end of treatment	No significant differences in BBS between groups were found
Lozano-Quilis et al. (41) JMIR Serious Games 2014	Spain	*n =* 5; 4 M/1 F Age: 40.6 ± 9.1 years	*n =* 6; 3 M/3 F Age: 48.3 ± 10.8 years	Not provided	Immersive VR, RemoviEM system, 10 sessions, 15 min, 1 time/week + Conventional balance training, 45 min	Conventional balance training 10 sessions, 60 min, 1 time/week	BBS at baseline and at the end of treatment	Significant differences in BBS between groups in favor of experimental group (*p* < 0.030)

*Values are presented as mean ± SD and mean (range)*.

*M, male; F, female; EDSS, Expanded Disability Status Scale; BBS, Berg Balance Scale*.

### Exergaming

Five RCTs (26, 36–39) assessed exergames as intervention compared with conventional balance training. Brichetto et al. (36) showed a significant improvement in the BBS in the experimental group after therapy (54.6 ± 2.2 vs. 49.7 ± 3.9; time × treatment: *p* < 0.05). Gutierrez et al. (37) reported a significant improvement in the BBS in the experimental group when comparing with control group at the end of the balance training (89.4 ± 6.6 vs. 81.9 ± 10.1; *F* = 29.896, *p* < 0.001). Similar results were found by Khalil et al. (26). They showed a significant difference between groups according to the balance score in favor of the experimental group (EG) (50.4 ± 3.7 vs. 45.1 ± 8.64; *p* = 0.012). On the other hand, Mohlemi et al. (38) investigated the efficacy of Xbox360^®^ plus conventional balance training vs. conventional rehabilitation, showing an improvement in the BBS in both the groups at the end of the treatment (EG: 46.6 ± 3.9 vs. 52.4 ± 2.1; *p* < 0.001; control group (CG) 45.5 ± 7.2 vs. 49.9 ± 5.5; *p* < 0.001) and at follow-up (52.0 ± 2.7; *p* < 0.001; 49.0 ± 5.7; *p* = 0.01, respectively). However, no differences between group were showed (*p* = 0.32 at the end; *p* = 0.10 at the follow-up). Similar results were found by Tollar et al. (39). The authors showed significant differences within groups in terms of balance activity after exergaming training (study group: 6.1 ± 3.5; *p* < 0.005 vs. control group: 3.9 ± 2.3; *p* < 0.005), but improvements in the BBS did not differ between groups.

### Virtual Reality

Two studies (40, 41) have investigated the effectiveness of VR vs. conventional balance training. Karlon et al. (40) in 2016 reported non-statistically significant differences between groups in the BBS score after treatment (47.9 ± 6.4 vs. 44.6 ± 4.9; *F* (*p*-value) = 1.794 (0.561)]. On the other hand, Lozano-Quilis et al. (41) used a kinect-based VR plus conventional balance training as intervention. A significant improvement in the BBS was found between groups in favor of the experimental group (50.3 ± 5.6 vs. 51.6 ± 5.8; *p* < 0.030).

### Meta-Analysis

**Figure 2:**
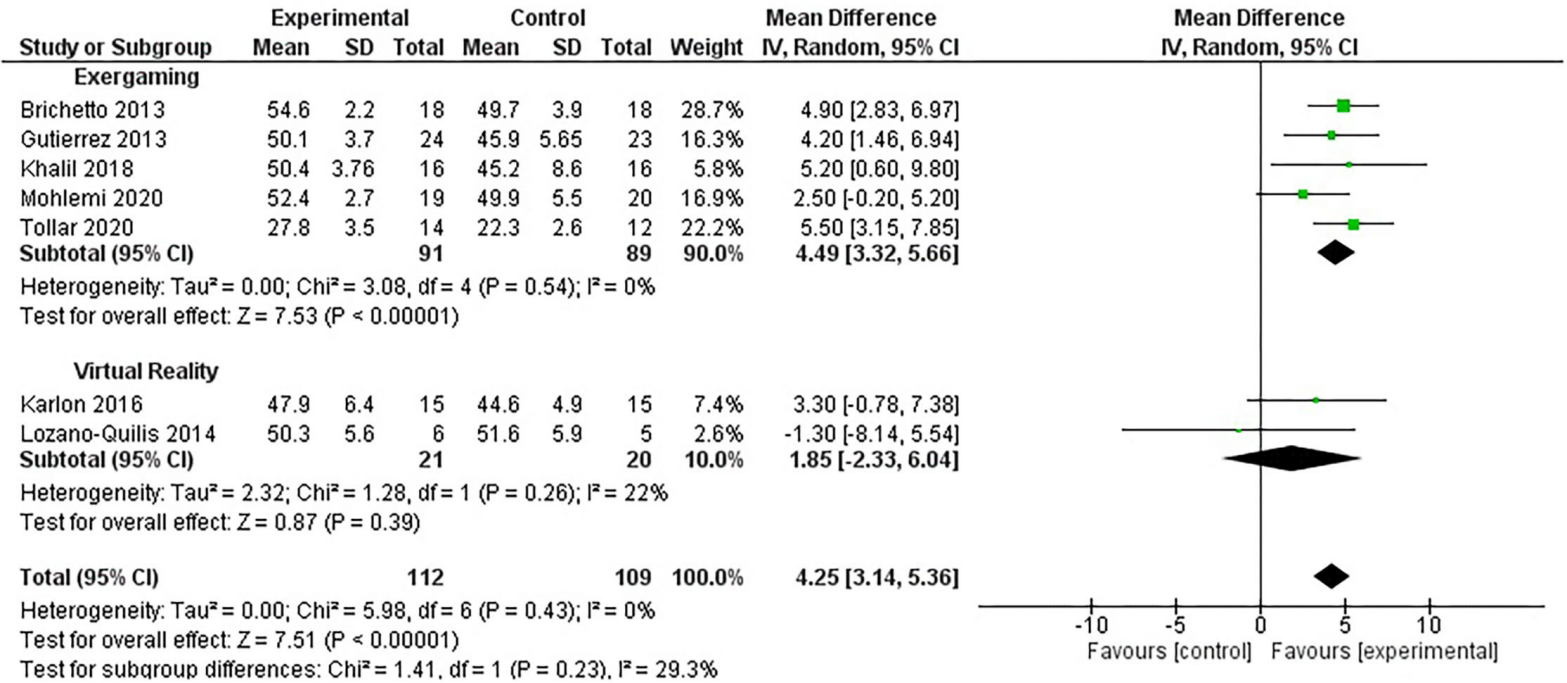
Forest plot illustrating the comparison between exergaming and virtual reality interventions vs. conventional rehabilitation through a meta-analysis.

**Figure 3:**
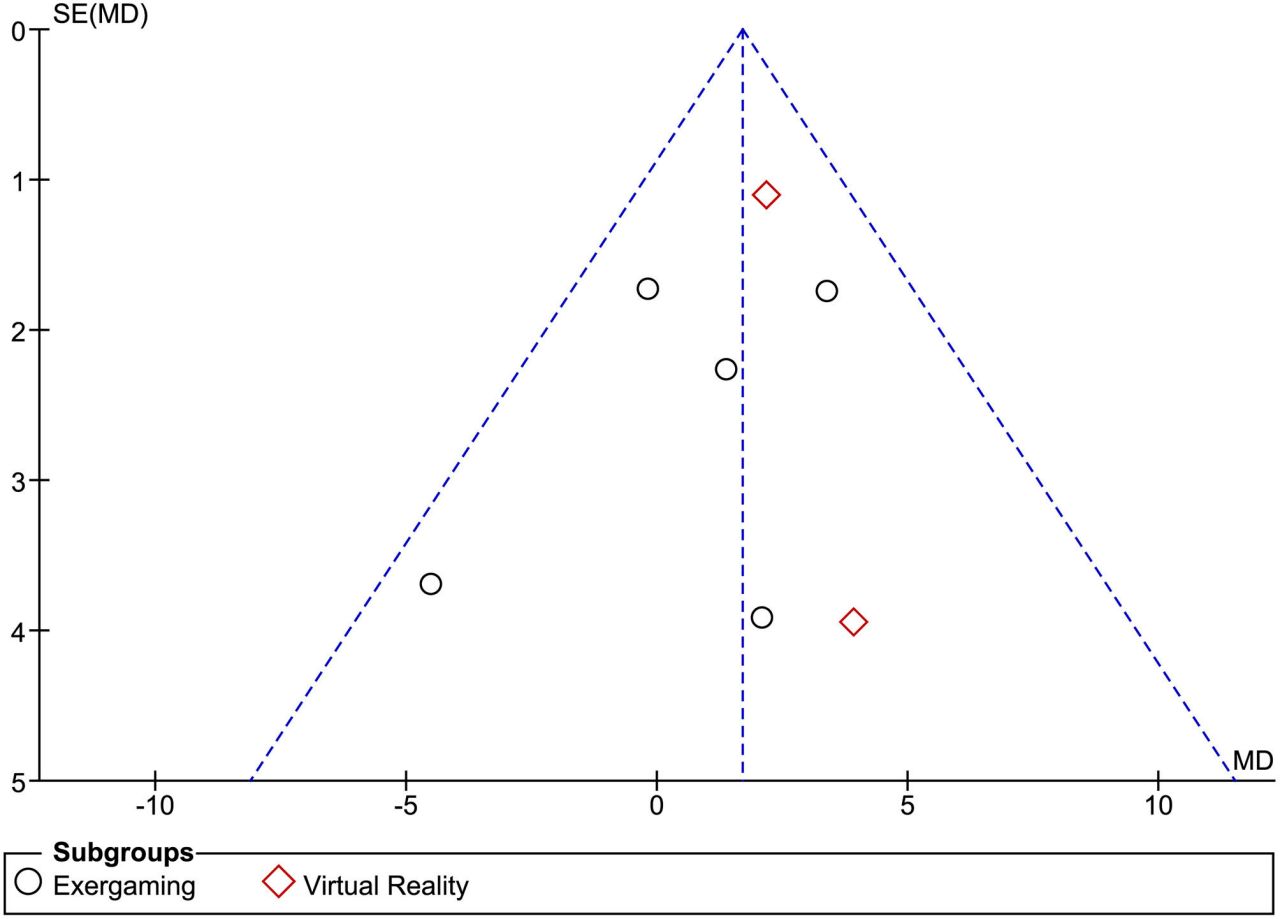
Begg's funnel plot analysis of publishing bias in the studies included in the present systematic review.

A meta-analysis was performed to highlight the efficacy of exergames and VR in improving balance (measured by the BBS) in patients affected by MS, showing an overall ES of 4.25 (95% CI = 3.14–5.36, *p* = 0.00001). The subgroup analysis reported a non-significant ES of VR in terms of the BBS improvement [1.85 (95% CI = 2.33–6.04), *p* = 0.39], whereas there was a significant improvement in the ES of the exergaming [4.49 (95% CI = 3.32–5.66), *p* = 0.00001], as shown by Figure 2. Given the low number of RCTs, a random-effects model was adopted. Moreover, the Begg's funnel plot analysis of publishing bias reported qualitatively symmetry in the RCTs included in this study, as shown in Figure 3.

### Risk-of-Bias

**Figure 4:**
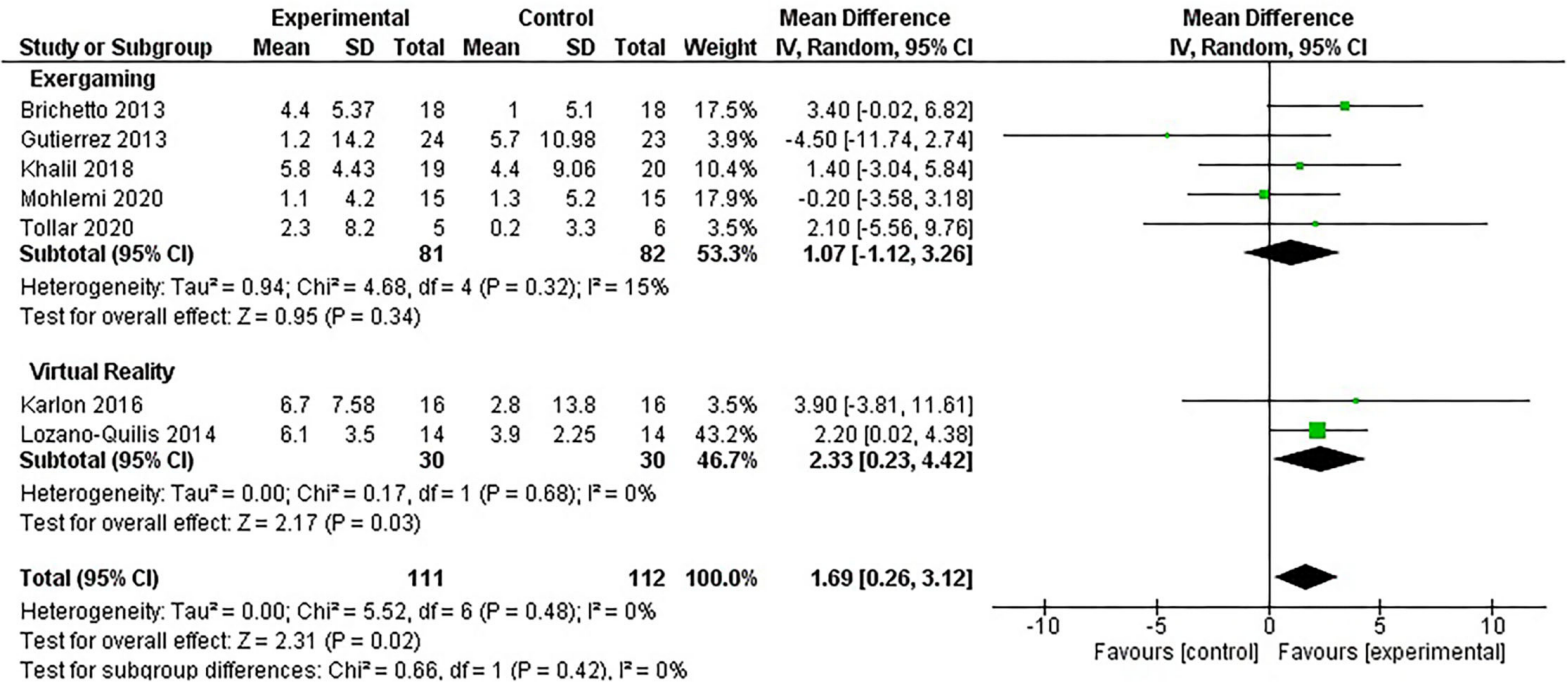
Risk-of-bias assessed by the version 2 of the Cochrane risk-of-bias 2 (RoB 2) tool for randomized trials.

The risk-of-bias among the RCTs analyzed was estimated using the RoB 2 (42) (see Figure 4 for further details). With respect to the selection bias, six studies (85.7%) ensured a correct randomization (26, 36–40). Five RCTs (71.4%) (26, 36–39) excluded performance bias. On the other hand, six studies (85.7%) (26, 36, 38–41) provided guarantees on blinding of outcome assessment and six studies (85.7%) (26, 36–40) adequately assessed attrition bias.

## Discussion

Virtual reality has recently emerged as a promising intervention in the rehabilitation of several neurological diseases (28–30, 43). This intriguing and complex technique can evoke brain behavioral responses that mimic real-world interaction, acting in real-time but in a safe environment. Exergaming consists of whole-body physical exercises comparable to a moderate intensity training, performed through active video games (44). It has been used in the rehabilitation of several neurological diseases to enhance both the cognitive and physical function and improve balance (45–47), as it offers task-oriented exercises enhancing motor learning and neural plasticity (48). Our findings are in line with previous evidence in the neurorehabilitation field, reporting that VR and exergaming are cumulatively effective on gait and balance in Parkinson's disease (49, 50), patients with poststroke (51), traumatic brain injury (52), and cerebral palsy (53). Despite the overall significance demonstrated for these two rehabilitation approaches (*p* = 0.00001), it should be noted that in the subgroup analysis, only exergames reported a significant effect size (*p* = 0.00001) compared to VR (*p* = 0.39).

Although robotic rehabilitation effects on balance and gait have been recently investigated with positive results in patients with MS (54), few studies in literature addressed the effectiveness of VR and exergames compared with conventional treatment in patients with MS and in most cases only considering VR as a complementary tool in MS rehabilitation concerning balance. This could be related to the relative novelty of these devices and the difficult implementation in the clinical setting.

Firstly, Casuso-Holgado et al. (55), analyzed in a systematic review the effectiveness of VR on gait and balance in patients affected by MS, showing significant differences in comparison with no interventions and inconclusive evidence compared with standard treatment. However, the authors included several different outcome measures (i.e., walking speed and postural balance). Moreover, Cano Porras et al. (56) in a systematic review found only three studies focusing on the BBS as primary outcome in patients with MS and VR rehabilitation, with inconclusive results.

Evidence on the role of VR in rehabilitative management of patients with MS is scarce, even though, in 2016, Massetti et al. (57) performed a systematic review on the effects of VR in patients affected by MS, including also observational studies and considering mixed outcomes. Although this approach widened the number of studies included, the low quality of the studies precluded to perform a meta-analysis. Furthermore, a recent meta-analysis performed by Nascimento et al. (58) suggested that VR could induce benefits that can be similar or greater than conventional exercises in patients with MS. However, taken together, all these studies were unable to draw strong conclusion about the real impact of VR on balance improvement in patients with MS, even though the effects of this approach are promising, considering the evidence obtained in other chronic neurological disorders (59, 60).

Concerning exergames, Mura et al. (45), found that in mixed neurological pathologies, including MS, they might significantly improve executive functions and visuospatial perception compared with no intervention or standard rehabilitative treatment. Concerning balance, successive studies in mixed neurological diseases confirmed that exergames might be at least equivalent to conventional therapy (59) and are able to improve balance dysfunction (60).

In the present systematic review and meta-analysis, we found that VR and exergaming might significantly improve balance in terms of the BBS compared with standard treatment alone in patients affected by MS. Among balance assessment, we assessed the BBS as primary outcome, since it is widely used and recommended in different neurological settings for patients with MS with EDSS ranging from 0 to 7.5 (61). Given that the esteemed minimal clinically important difference for the BBS is 3 points (62), most of the selected studies showed a clinically significant difference between standard treatment and exergaming/VR interventions. Furthermore, VR and exergames might improve balance proposing simultaneous motor and cognitive tasks (63) that might also be addictive, improving both the motivation and treatment adherence (64). In this study, repetitive practice and observation are crucial for motor learning and VR might induce plastic changes in central nervous system that has been associated with mirror imagery in other neurological disorders such as stroke survivors through a facilitation effect on sensorimotor networks (65). The high adherence observed in patients performing exergaming might be due to the low practical barriers, high accessibility, low cost of the consoles, and the social impact because of the potential involvement of family members in multiplayer games (21). Moreover, exergames provide visual and auditory feedback, currently altered in patients affected by MS (66), thus improving the self-awareness of the patients during the training. Furthermore, Yazgan et al. (67) demonstrated a significant improvement in terms of fatigue and gait after exergaming treatment. The authors suggested that these improvements were obtained thanks to the less anxiety and greater confidence in the balance raised by the videogame approach and not a low energy expenditure compared to standard treatments.

This systematic review and meta-analysis have also some clinical implications for the rehabilitation clinical practice, considering that VR resulted to be an intriguing alternative for balance training in patients with MS, with psychological advantages that could enhance their motivation and treatment adherence (68). Clinicians should strike the right balance between too difficult and too easy tasks and as such keeping the motivation of the patients high. Objective progression and extrinsic feedback encourage robot-assisted rehabilitation that might play a critical role on neuroplasticity (69, 70). Lastly, it should be considered that VR might be home based, with a telerehabilitation approach, which is highly encouraged during coronavirus disease 2019 (COVID-19) pandemic, due to psychological and hospitalization issues (71–73).

We are aware that our systematic review considered only a small number of RCTs due to the limited evidence available in the literature. Hence, further high-quality studies investigating exergames and VR effects in improving balance in patients with MS compared with conventional rehabilitation treatment are still warranted and the use of relatively recent exergaming devices is not created specifically for neurorehabilitation. Moreover, to improve the strength of evidence on VR, future RCTs addressing this specific issue in patients with MS are warranted.

## Conclusion

This study suggested that rehabilitative interventions using exergames and VR appear to be more effective than conventional rehabilitation to improve balance in patients with MS. More in detail, exergames showed to have a significant efficacy in improving balance outcomes and considering its safety and its effects on neuroplasticity, sensorimotor training, and motivation of the patients, it could be implemented as an effective technique in the complex rehabilitative treatment framework of neurological diseases including MS. Starting from these promising data, further evidence is warranted in the next future to focus on VR and its role in the rehabilitative approach to neurological disorders.

## Data Availability Statement

The raw data supporting the conclusions of this article will be made available by the authors, without undue reservation.

## Author Contributions

DC and AS contribute to the study design and conceptualization. AS contributes to the databases searching. DC, CC, and AC-S contribute to the data screening. AA, FFo, and AS contribute to the data extraction. DC, MI, and AS contribute to the data synthesis and interpretation. NM contributes to the statistical analysis. DC contributes to the manuscript drafting. MI and AS contribute to the critical revision, study supervision, and study submission. AA, NM, FFo, TP, FFe, CC, and AC-S contribute to the visualization. All the authors read and approved the final version of the manuscript.

## Conflict of Interest

The authors declare that the research was conducted in the absence of any commercial or financial relationships that could be construed as a potential conflict of interest.

## Publisher's Note

All claims expressed in this article are solely those of the authors and do not necessarily represent those of their affiliated organizations, or those of the publisher, the editors and the reviewers. Any product that may be evaluated in this article, or claim that may be made by its manufacturer, is not guaranteed or endorsed by the publisher.
