# Supplementary material for: Efficacy of Virtual Reality and Exergaming in Improving Balance in Patients With Multiple Sclerosis: A Systematic Review and Meta-Analysis
Source: Front Neurol. 2021 Dec 10;12:773459. doi: 10.3389/fneur.2021.773459 (PMC8702427; doi:10.3389/fneur.2021.773459)
Supplement: Supplementary file 1 [file Table_1.DOCX]

| **Supplementary Table 1.** Search strategy. |
| --- |
| ***PubMed***  (“multiple sclerosis”) AND ("virtual reality" OR "exergames" OR "exergaming" OR "home gaming" OR “videogame” OR “video game”) AND ("balance" OR “postural balance” OR “posture” OR “Berg Balance Scale”) |
| ***Scopus***  TITLE-ABS-KEY(((“multiple sclerosis”) AND ("virtual reality" OR "exergames" OR "exergaming" OR "home gaming" OR “videogame” OR “video game”) AND ("balance" OR “postural balance” OR “posture” OR “Berg Balance Scale”))) |
| ***Web of Science***  ((“multiple sclerosis”) AND ("virtual reality" OR "exergames" OR "exergaming" OR "home gaming" OR “videogame” OR “video game”) AND ("balance" OR “postural balance” OR “posture” OR “Berg Balance Scale”)) |
